# Lack of Cerebrospinal Fluid α‐Synuclein Seeding in *VPS35* D620N‐ and *LRRK2* Y1699C‐Linked Parkinson's Disease

**DOI:** 10.1002/mds.70291

**Published:** 2026-03-30

**Authors:** Letizia Santinelli, Neringa Pratuseviciute, Lara M. Lange, Teresa Kleinz, Norbert Brüggemann, Linn Streubel‐Gallasch, Meret Möller, Alexander Zimprich, Christof Brücke, Jean‐Marc Good, Norman Griebner, Shalini Padmanabhan, Philip Seibler, Peter Bauer, Wim Vandenberghe, Esther Sammler, Cornelis Blauwendraat, Christine Klein, Max Borsche

**Affiliations:** ^1^ Institute of Neurogenetics University of Lübeck Lübeck Germany; ^2^ MRC Protein Phosphorylation and Ubiquitylation Unit, School of Life Sciences University of Dundee Dundee United Kingdom; ^3^ Section for Movement Disorders, Department of Neurology University of Lübeck Lübeck Germany; ^4^ Laboratory of Neurogenetics National Institute on Aging Bethesda Maryland USA; ^5^ Comprehensive Center for Clinical Neurosciences and Mental Health Medical University of Vienna Vienna Austria; ^6^ Department of Neurology Medical University Vienna Vienna Austria; ^7^ Division of Genetic Medicine Lausanne University Hospital and University of Lausanne Lausanne Switzerland; ^8^ The Michael J. Fox Foundation for Parkinson's Research New York New York USA; ^9^ CENTOGENE GmbH Rostock Germany; ^10^ Pomeranian Medical University Szczecin Poland; ^11^ Laboratory for Parkinson Research Department of Neurosciences Leuven Belgium; ^12^ Department of Neurology University Hospitals Leuven Leuven Belgium; ^13^ Molecular and Clinical Medicine Ninewells Hospital and Medical School, University of Dundee Dundee United Kingdom; ^14^ National Institutes of Health, Center for Alzheimer's and Related Dementias National Institute on Aging and National Institute of Neurological Disorders and Stroke Bethesda Maryland USA

**Keywords:** α‐synuclein, biomarker, kinase activity, *LRRK2* Y1699C, monogenic Parkinson's disease, SAA, seed amplification assay, *VPS35* D620N

α‐Synuclein's (α‐syn) aggregation properties enabled the development of seed amplification assays (SAA), accurately distinguishing between idiopathic Parkinson's disease (PD) patients and healthy controls in cerebrospinal fluid (CSF) analyses.^1^ Among autosomal dominant PD in European ancestry populations, *LRRK2* pathogenic variants are the most frequent (~2.6%), whereas *VPS35*‐linked PD occurs in <0.06% of PD patients.^2^ LRRK2 and VPS35 are key regulators of intracellular transport.^3^ *LRRK2*‐linked PD exhibits heterogeneous α‐syn pathology as Lewy body pathology and CSF‐SAAs are positive only in approximately two‐thirds of patients.^1^, ^4^ Postmortem data from the single reported *VPS35*‐linked PD patient revealed no typical Lewy body pathology.^4^ Different variants in the *LRRK2* gene affect kinase activity to varying degrees, and the rare PD‐causing variant Y1699C is associated with extensive kinase activation in vitro.^5^ Of note, the only known pathogenic *VPS35* D620N variant also increases LRRK2 kinase activity.^6^

No prior SAA studies exist for the *LRRK2* Y1699C and the *VPS35* D620N variants. We performed CSF‐SAA (Supplementary Methods) in 3 PD patients carrying the *VPS35* D620N variant and 2 *LRRK2* Y1699C variant carriers (1 affected, 1 unaffected) (Supplementary Clinical Information, Fig. S1). Strikingly, neither the affected *VPS35* D620N‐linked PD patients nor the *LRRK2* Y1699C variant carriers were CSF‐SAA positive (Table 1), even though the investigated *LRRK2* family members carried an additional *GBA1* E365K variant.

**Table 1:** Demographics, PD diagnosis, clinical characteristics, DaTSCAN, and SAA results.

ID	L‐14826	L‐15868	L‐27026	L‐19059	L‐20698
	Family 1	Family 1	Family 2	Family 3	Family 3
Genetics					
Genetic variant	*VPS35* D620N			*LRRK2* Y1699C *GBA1* E365K	
Demographics					
Sex	Female	Male	Female	Male	Male
AAE (years)	70	71	65	57	36
Ethnicity	White	White	White	White	White
Parkinson's disease diagnosis and motor signs					
PD diagnosis	Yes	Yes	Yes	Yes	No
Bradykinesia	Yes	Yes	Yes	Yes	No
Rigidity	Yes	Yes	Yes	Yes	No
Resting tremor	No	No	Yes	No	No
Postural instability	No	No	No	No	No
m‐EDL (MDS UPDRS part II)	13	12	7	9	0
MDS‐UPDRS part III (“ON”)	18	20	30	14	0
Dyskinesia	Yes	No	No	Yes	No
Motor fluctuations	Yes	Yes	No	Yes	No
MDS‐UPDRS part IV	9	3	0	8	0
Dystonia	No	No	No	Yes	No
Atypical signs	No	No	No	No	No
Disease onset, duration, and state					
AAO (years)	58	57	52	49	NA
Disease duration (years)	12	14	13	8	NA
Hoehn and Yahr stage	2	2	2	2	0
Non‐motor signs					
nm‐EDL (MDS UPDRS part I)	16	12	12	8	1
Hyposmia	Yes	Yes	No	No	No
BSIT/UPSIT items correct/age‐ and sex‐matched percentile	13/40, 8th	11/40, 5th	30/40, 19th	9/12, 29th	10/12, 23rd
Cognitive impairment	Yes	No	No	No	No
MoCA items correct	24/30	27/30	29/30	27/30	29/30
Technical measures					
DaTSCAN	Abnormal	Abnormal	Abnormal	NA	NA
SAA	Negative	Negative	Negative	Negative	Negative
Kinase activity data available	Yes	Yes	No	Yes	Yes

*Notes*: Hyposmia was determined by the BSIT (Brief Smell Identification Test)/UPSIT (University of Pennsylvania Smell Identification Test), defined by a score below the age‐ and sex‐dependent 10th percentile; Cognitive impairment was assessed by MoCA (Montreal Cognitive Assessment) testing, considering a score equal/below 26 as (mild) cognitive impairment. Details on recruitment, genetic testing and phenotyping are depicted in Supplementary Methods, details on the included individuals are provided in Supplementary Clinical Information, and pedigrees of Family 1 and 3 are shown in Figure S1.

Abbreviations: AAE, age at examination; PD diagnosis, confirmed diagnosis of Parkinson's disease according to the MDS (Movement Disorder Society) criteria; m‐EDL, Motor Experiences of Daily Living; MDS‐UPDRS, Movement Disorders Society Unified Parkinson's Disease Rating Scale; Atypical signs, Presence of atypical parkinsonian features according to the Movement Disorders Society criteria; AAO, age at onset; NA, not available; nm‐EDL, non‐motor Experiences of Daily Living.

Our results in *LRRK2* Y1699C variant carriers align with the reported atypical postmortem findings, and both were normosmic, in keeping with the established association between preserved olfaction and SAA negativity in *LRRK2*‐linked PD.^1^ By contrast, 2 of 3 *VPS35*‐linked PD patients were anosmic yet showed negative SAA results (Table 1), potentially indicating that anosmia in *VPS35*‐linked PD may not be associated with α‐syn pathology. Together, SAA negativity in *VPS35* D620N and *LRRK2* Y1699C variant carriers may suggest that further mechanisms beyond the shared autophagic‐lysosomal pathway potentially influence α‐syn aggregation detectable by SAA. Within the framework of the recently proposed attempts to biologically define PD, particularly *VPS35*‐linked PD would not fulfill the criteria for neuronal α‐syn disease defined by the NSD‐ISS (Neuronal α‐Synuclein Disease Integrated Staging System) classification according to our data.^7^

To complement SAA analyses, we quantified in vivo LRRK2 kinase activity in neutrophils (Supplementary Methods, Table S1), revealing significant increases in *VPS35* D620N (about threefold, n = 6) and *LRRK2* Y1699C variant carriers (about eightfold, n = 3) compared to healthy controls (Fig. 1; Fig. S2). Thus, our findings suggest that LRRK2 kinase hyperactivation does not necessarily determine SAA positivity. Particularly, we confirmed that the *LRRK2* Y1699C variant elevates kinase activity in vivo, extending previous in vitro observations.^5^

**FIG. 1:**
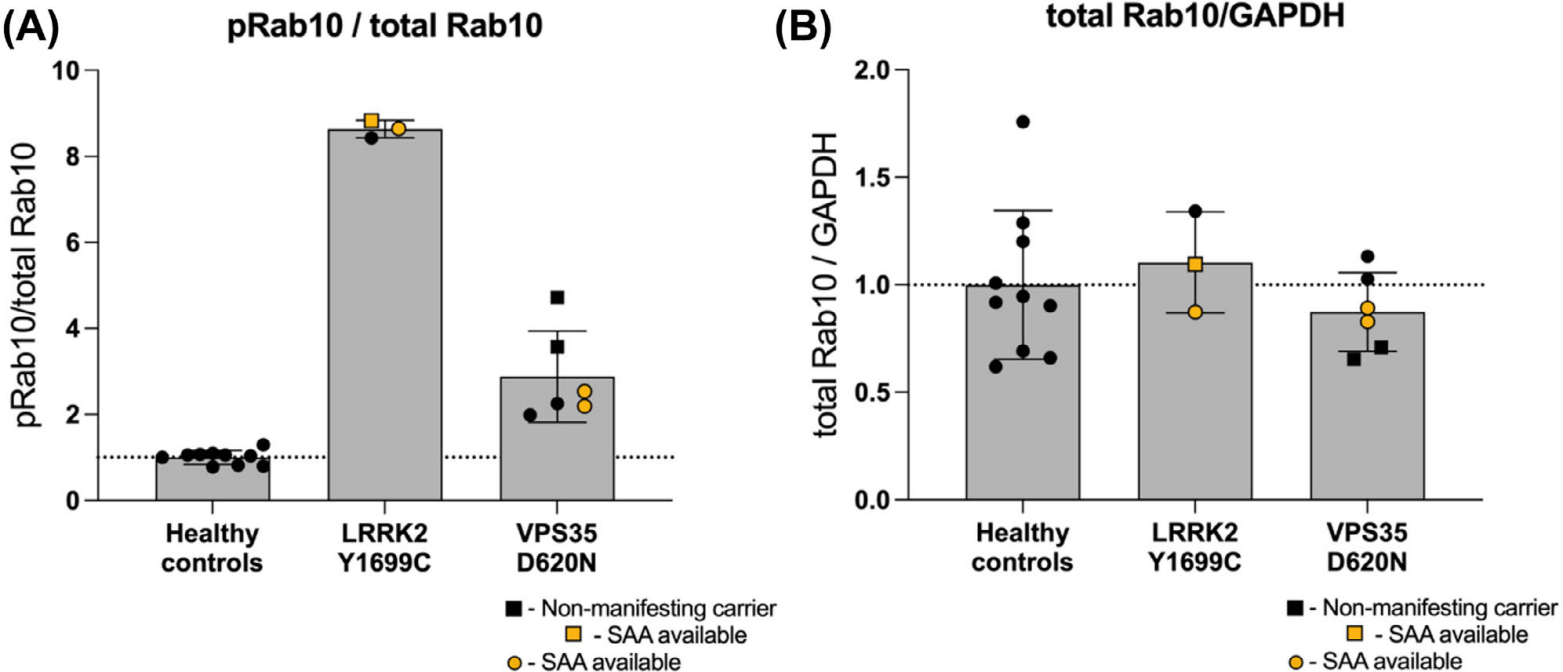
LRRK2‐dependent Rab10^Thr73^ phosphorylation in *LRRK2* Y1699C and *VPS35* D620N variant carriers. Neutrophils were isolated from ~10 mL of fresh peripheral blood from *VPS35* D620N variant carriers (n = 6), *LRRK2* Y1699C variant carriers (n = 3), and healthy controls. After cell lysis, the samples were analyzed using quantitative immunoblot analysis. Quantified results for all samples were normalized to the mean of all healthy controls (*y* = 1) and expressed as (**A**) pRab10/total Rab10 and (**B**) total Rab10/GAPDH. PD (Parkinson's disease) patients are represented by circles, and nonmanifesting carriers are represented by squares. Yellow items refer to patients with SAA (seed amplification assay) data. Western blots underlying the data in panels A and B are shown in Figure S2.

In summary, our study underscores the potential of SAAs to disentangle the mechanisms underlying rare monogenic forms of PD. The apparent dissociation between pronounced LRRK2 kinase activation and α‐syn deposition may have key implications for patient stratification and the design of disease‐modifying trials. Due to the rarity of these genetic PD subtypes, further research will require multicenter efforts to assemble larger samples and clarify the underlying pathophysiology.

## Author Roles

(1) Research Project: A. Conception, B. Organization, C. Execution; (2) Statistical Analysis: A. Design, B. Execution, C. Review and Critique; (3) Manuscript Preparation: A. Writing of the First Draft, B. Review and Critique.

L.S.: 1A, 1B, 1C, 2A, 2B, 3A.

N.P.: 1B, 1C, 2C, 3B.

L.M.L.: 1C, 2C, 3B.

T.K.: 1C, 2C, 3B.

N.B.: 1C, 2C, 3B.

L.S.‐G.: 1B, 1C, 2C, 3B.

M.M.: 1C, 2C, 3B.

A.Z.: 1B, 1C, 2C, 3B.

C.B.: 1C, 2C, 3B.

J.‐M.G.: 1B, 1C, 2C, 3B.

N.G.: 1C, 2C, 3B.

S.P.: 1A, 1B, 2C, 3B.

P.S.: 1B, 1C, 2C, 3B.

P.B.: 1B, 1C, 2C, 3B.

W.V.: 1B, 1C, 2C, 3B.

E.S.: 1A, 1B, 1C, 2C, 3B.

C.B.: 1A, 1B, 1C, 2C, 3B.

C.K.: 1A, 1B, 1C, 2C, 3B.

M.B.: 1A, 1B, 1C, 2A, 2B, 3A.

The contributions of the NIH author(s) were made as part of their official duties as NIH federal employees, are in compliance with agency policy requirements, and are considered Works of the United States Government. However, the findings and conclusions presented in this paper are those of the author(s) and do not necessarily reflect the views of the NIH or the U.S. Department of Health and Human Services.

## Financial Disclosures and Conflicts of Interest

Author disclosures are available in the Supporting Information.

## Supporting information


**Data S1:** Comprises Supplementary Methods, Supplementary Clinical Information, Table S1 (Overview on additional individuals included in LRRK2 kinase activity analyses), Figure S1 (Pedigrees of Family 1 and 3), Figure S2 (Representative immunoblots demonstrating LRRK2 dependent Rab10Thr73phosphorylation in patient‐ and control‐derived clinical samples), and Supplementary References.

## Data Availability

The data that support the findings of this study are available from the corresponding author upon reasonable request.

## References

[1] Siderowf A , Concha‐Marambio L , Lafontant DE , et al. Assessment of heterogeneity among participants in the Parkinson's progression markers initiative cohort using α‐synuclein seed amplification: a cross‐sectional study. Lancet Neurol 2023;22:407–417. 37059509 10.1016/S1474-4422(23)00109-6 PMC10627170

[2] Westenberger A , Skrahina V , Usnich T , et al. Relevance of genetic testing in the gene‐targeted trial era: the Rostock Parkinson's disease study. Brain 2024;147:2652–2667. 39087914 10.1093/brain/awae188 PMC11292909

[3] Alessi DR , Cullen PJ , Cookson M , Merchant KM , Small SA . Retromer‐dependent lysosomal stress in Parkinson's disease. Philos Trans R Soc Lond B Biol Sci 2024;379:20220376. 38368937 10.1098/rstb.2022.0376 PMC10874697

[4] Schneider SA , Alcalay RN . Neuropathology of genetic Synucleinopathies with parkinsonism – review of the literature. Mov Disord 2017;32:1504–1523. 29124790 10.1002/mds.27193 PMC5726430

[5] Kalogeropulou AF , Purlyte E , Tonelli F , et al. Impact of 100 LRRK2 variants linked to Parkinson's disease on kinase activity and microtubule binding. Biochem J 2022;479:1759–1783. 35950872 10.1042/BCJ20220161 PMC9472821

[6] Mir R , Tonelli F , Lis P , et al. The Parkinson's disease VPS35[D620N] mutation enhances LRRK2‐mediated Rab protein phosphorylation in mouse and human. Biochem J 2018;475:1861–1883. 29743203 10.1042/BCJ20180248 PMC5989534

[7] Simuni T , Chahine LM , Poston K , et al. A biological definition of neuronal α‐synuclein disease: towards an integrated staging system for research. Lancet Neurol 2024;23:178–190. 38267190 10.1016/S1474-4422(23)00405-2

