# Supplementary material for: Lack of Cerebrospinal Fluid α‐Synuclein Seeding in VPS35 D620N‐ and LRRK2 Y1699C‐Linked Parkinson's Disease
Source: Mov Disord. 2026 Mar 30;41(6):1605–8. doi: 10.1002/mds.70291 (PMC13307260; doi:10.1002/mds.70291)
Supplement: Supplementary file 1 — Data S1: Comprises Supplementary Methods, Supplementary Clinical Information, Table S1 (Overview on additional individuals included in LRRK2 kinase activity analyses), Figure S1 (Pedigrees of Family 1 and 3), Figure S2 (Representative immunoblots demonstrating LRRK2 dependent Rab10Thr73phosphorylation in patient‐ and control‐derived clinical samples), and Supplementary References. [file MDS-41-1605-s001.docx]

**Supplementary material for “Lack of CSF alpha-synuclein seeding in *VPS35* D620N- and *LRRK2* Y1699C-linked Parkinson's disease”**

**Supplementary Methods:**

Parkinson’s disease (PD) patients from Families 1 and 3 were identified within the ROPAD study,^1^ and one additional unaffected variant carrier from Family 3 was enrolled in this study. One PD patient with the *VPS35* D620N variant is a member of a recently identified Flemish family (Family 2) described elsewhere.^2^ All participants were invited to the University of Lübeck for deep phenotyping and biomaterial collection. The PD diagnosis was established according to the MDS criteria.^3,4^ DaTSCAN imaging data were available for all *VPS35*-linked PD patients with SAA data. The DaTSCANs in Family 1 were performed according to the PPMI protocol,^5^ while the Flemish patient received a DaTSCAN as part of clinical diagnostics. Genetic testing in Family 1 and 3 was performed as described.^1^ While the unaffected *LRRK2* Y1699C variant carrier underwent ROPAD panel analyses at CENTOGENE, the Flemish patient was tested with a diagnostic panel including the *VPS35* variant after detection in her family members.^1^

CSF samples were processed in Lübeck according to the PPMI protocol,^6^ frozen at -80°C, and shipped temperature-controlled to Amprion. CSF samples were analyzed in a blinded fashion as previously described,^7^ yielding dichotomous results regarding CSF α-syn pathology (SAA-positive vs. SAA-negative) for each participant.

Regarding *in vivo* LRRK2 kinase activity analyses, blood taking, neutrophil isolation via immunomagnetic negative selection, and multiplexed quantitative immunoblot analyses were performed as described.^8^ The Ethics Committee of the University of Lübeck, Germany, approved the study (Az. 16-039), and all participants gave written informed consent.

**Supplementary Clinical Information:**

Family 1 (Figure S1A) is from the Northeast of Germany. The *VPS35* D620N variant was detected in the index patient (III.2), a 70-year-old White female who developed first PD symptoms at the age of 58 years. Her cousin (III.5), a 71-year-old White male PD patient with an age at onset of 57 years, also carries the *VPS35* D620N variant. Both had hyposmia and abnormal DaTSCAN imaging according to visual and semiquantitative analyses.

The third patient with the *VPS35* D620N variant belongs to a recently described Flemish family (Family 2).^2^ She is a 65-year-old White female who developed first symptoms of PD at 52 years of age. She had no hyposmia. A DaTSCAN showed pathological results.

Family 3 (Figure S1B) is from Northern Germany and includes two family members carrying the *LRRK2* Y1699C variant. The index patient (III.1) is a 57-year-old White male who developed first symptoms at the age of 49 years. His sense of smell was unimpaired. In addition to the *LRRK2* Y1699C variant, he carries the *GBA1* E365K variant. PD patients negative for the *LRRK2* Y1699C and the *GBA1* E365K variant are also present in the family (II.6, III.12). Besides the index patient, his son (IV.2), who carries the same variants, also donated CSF. At examination, he was 36 years old and had no motor or non-motor PD signs or symptoms. In all three families, individuals with PD were present in every generation, in keeping with autosomal dominant inheritance (Figure S1).

**Supplementary Tables and Figure:**

| **ID** | **Group** | **Genetic variant** | Sex | AAE (years) | AAO (years) |
| --- | --- | --- | --- | --- | --- |
| L-26780 | Healthy control | NA | Male | 71 | NA |
| L-25738 | Healthy control | NA | Male | 33 | NA |
| L-20609 | Healthy control | NA | Male | 35 | NA |
| L-25543 | Healthy control | NA | Male | 53 | NA |
| L-25739 | Healthy control | NA | Female | 27 | NA |
| L-8772 | Healthy control | NA | Male | 59 | NA |
| L-8649 | Healthy control | NA | Female | 58 | NA |
| L-6774 | Healthy control | NA | Female | 67 | NA |
| L-24753 | Healthy control | NA | Female | 58 | NA |
| L-20604 | LRRK2 PD | *LRRK2* Y1699C  *GBA1* E365K | Male | 73 | 60 |
| L-25544 | VPS35 PD | *VPS35* D620N | Female | 52 | 47 |
| L-24752 | VPS35 NMC | *VPS35* D620N | Male | 55 | NA |
| L-24754 | VPS35 NMC | *VPS35* D620N | Female | 34 | NA |

**Table S1:** **Overview on additional individuals included in LRRK2 kinase activity analyses.** The genetic variant, sex and age at examination/blood taking are shown. NA – not available. PD – Parkinson’s disease patient. NMC – Non-manifesting variant carrier. Information on individuals with both SAA and kinase activity data are shown in Table 1.

**
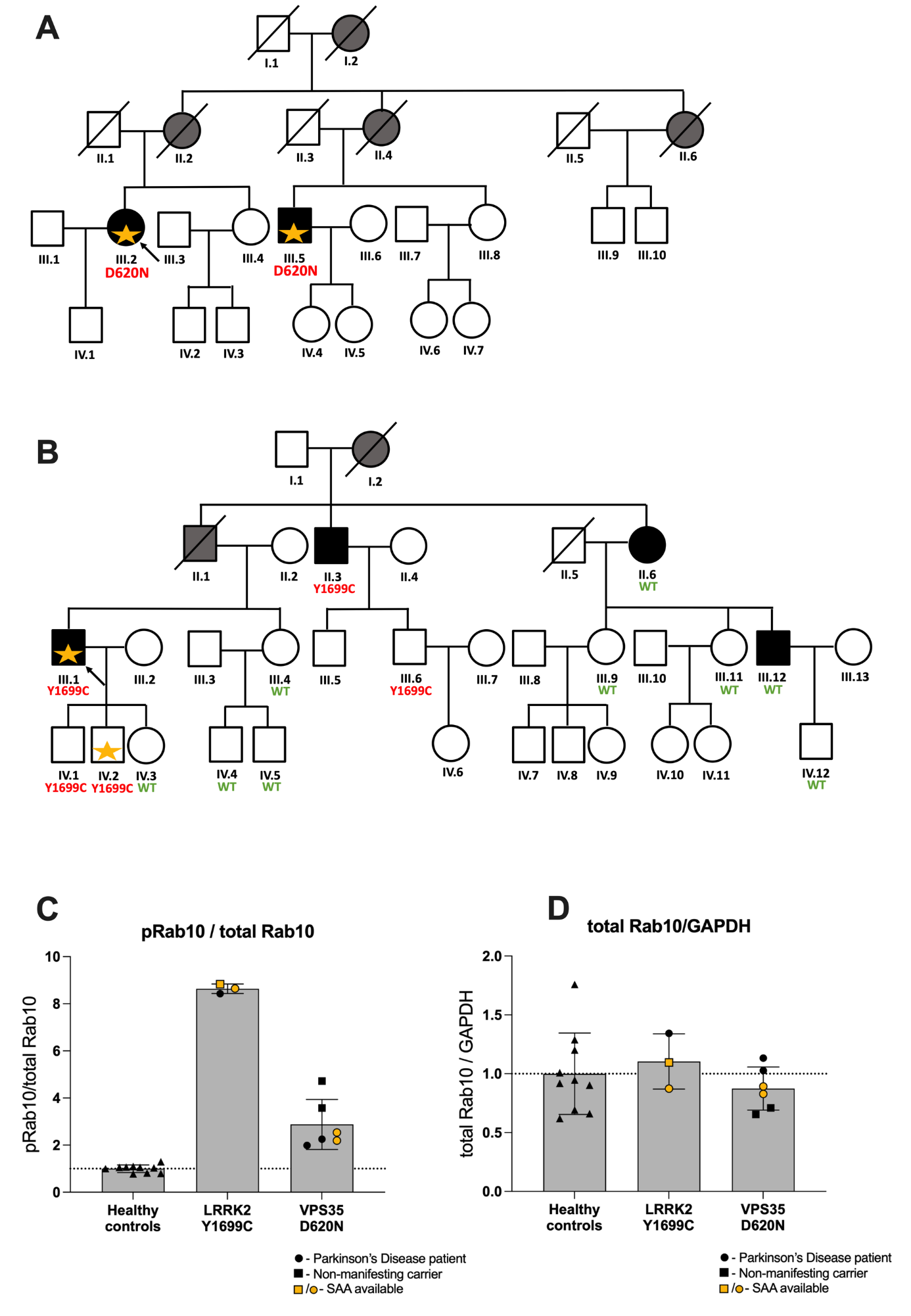
**

**Figure S1: Pedigrees of Family 1 and 3.** Circle = female, square = male, diamonds = unspecific sex, number in the diamond refers to the number of children; strikethrough = deceased; black = diagnosis of PD based on clinical examination by the study team; grey = PD reported; variants occurring in the family are shown in red; WT = negative genetic testing for the variant present in the family; yellow star = CSF available and α-synuclein seed amplification assay (SAA) analyses performed; arrow = index patient. **A.** Family 1 from Northeast Germany with family members carrying the *VPS35* D620N variant. **B.** Family 3 from Northern Germany, with family members carrying the *LRRK2* Y1699C variant. Unaffected variant carriers (III.6, IV.1, and IV.2) were below the age of 40 at examination.


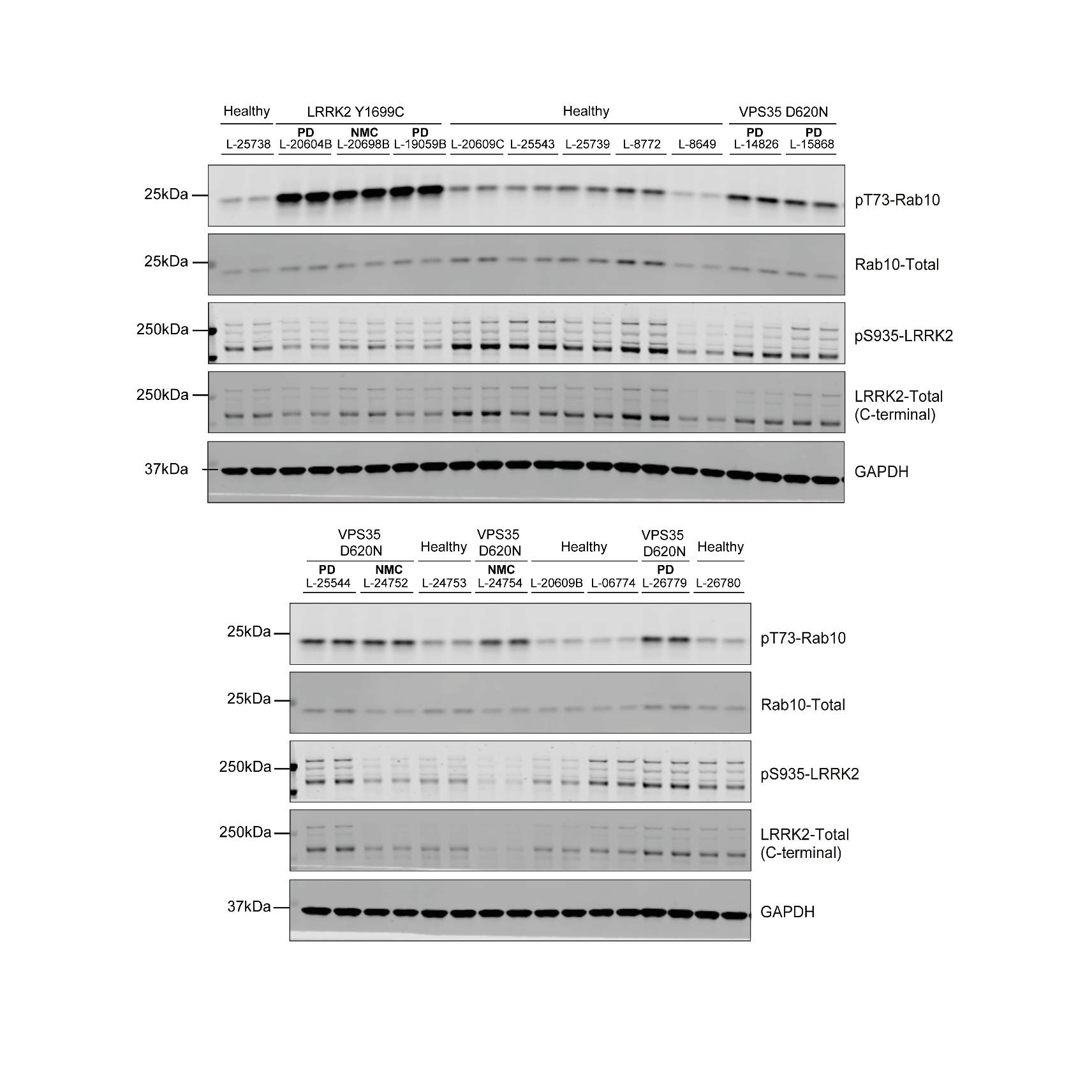


**Figure S2:** **Representative immunoblots demonstrating LRRK2 dependent Rab10^Thr73^phosphorylation in patient- and control-derived clinical samples.** Neutrophils were isolated from indicated heterozygous *VPS35* and *LRRK2* variant carriers and healthy controls. 10µg of each sample was loaded for Western Blot analysis; membranes were thereafter incubated with indicated antibodies and imaged using LICOR Odyssey CLx imaging system.

**Supplementary References:**

1. Westenberger A, Skrahina V, Usnich T, Beetz C, Vollstedt EJ, Laabs BH, et al. Relevance of genetic testing in the gene-targeted trial era: the Rostock Parkinson’s disease study. Brain. 2024;147:2652–67.

2. Manders L, Heyninck T, Imberechts D, Holst B, Krüger R, Vandenberghe W. VPS35 mutation inhibits PINK1/parkin-mediated mitophagy via increased LRRK2 kinase activity. Brain. 2025;awaf414.

3. Postuma RB, Berg D, Stern M, Poewe W, Olanow CW, Oertel W, et al. MDS clinical diagnostic criteria for Parkinson’s disease. Mov Disord. 2015;30:1591–601.

4. Postuma RB, Poewe W, Litvan I, Lewis S, Lang AE, Halliday G, et al. Validation of the MDS clinical diagnostic criteria for Parkinson’s disease. Mov Disord. 2018;33:1601–8.

5. Marek K, Chowdhury S, Siderowf A, Lasch S, Coffey CS, Caspell-Garcia C, et al. The Parkinson’s progression markers initiative (PPMI) – establishing a PD biomarker cohort. Ann Clin Transl Neurol. 2018;5:1460–77.

6. Siderowf A, Concha-Marambio L, Lafontant DE, Farris CM, Ma Y, Urenia PA, et al. Assessment of heterogeneity among participants in the Parkinson’s Progression Markers Initiative cohort using α-synuclein seed amplification: a cross-sectional study. Lancet Neurol. 2023;22:407–17.

7. Concha-Marambio L, Pritzkow S, Shahnawaz M, Farris CM, Soto C. Seed amplification assay for the detection of pathologic alpha-synuclein aggregates in cerebrospinal fluid. Nat Protoc. 2023;18:1179–96.

8. Fan Y, Howden AJM, Sarhan AR, Lis P, Ito G, Martinez TN, et al. Interrogating Parkinson’s disease LRRK2 kinase pathway activity by assessing Rab10 phosphorylation in human neutrophils. Biochem J. 2018;475:23–44.
